# Supplementary figures and images for: Proteomics-based screening of AKR1B1 as a therapeutic target and validation study for sepsis-associated acute kidney injury
Source: PeerJ. 2024 Jan 2;12:e16709. doi: 10.7717/peerj.16709 (PMC10768659; doi:10.7717/peerj.16709)

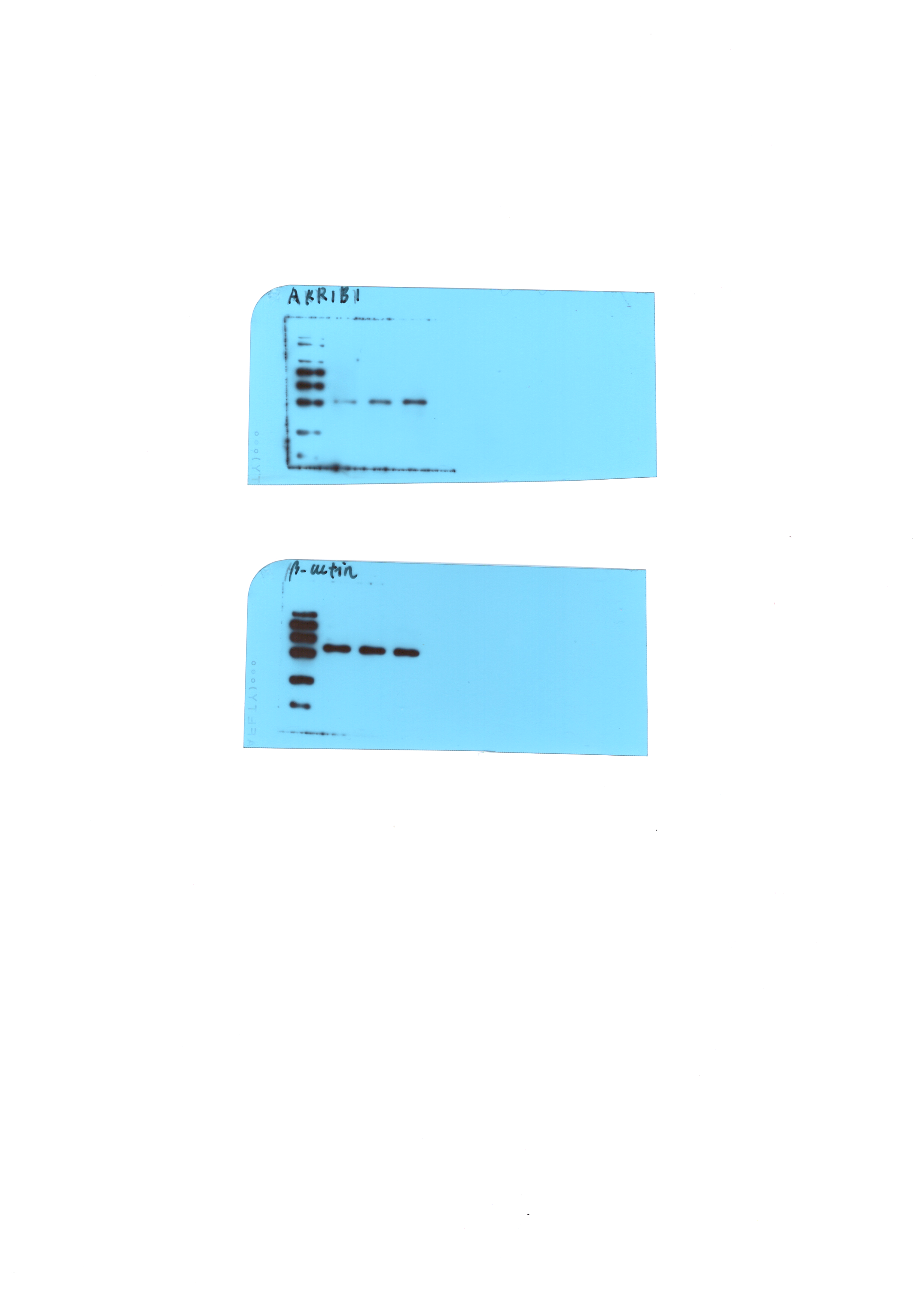

Supplement: Data S1 [file peerj-12-16709-s001.zip › Raw data/Figure 2/Figure 2A/Figure 2A.tif]

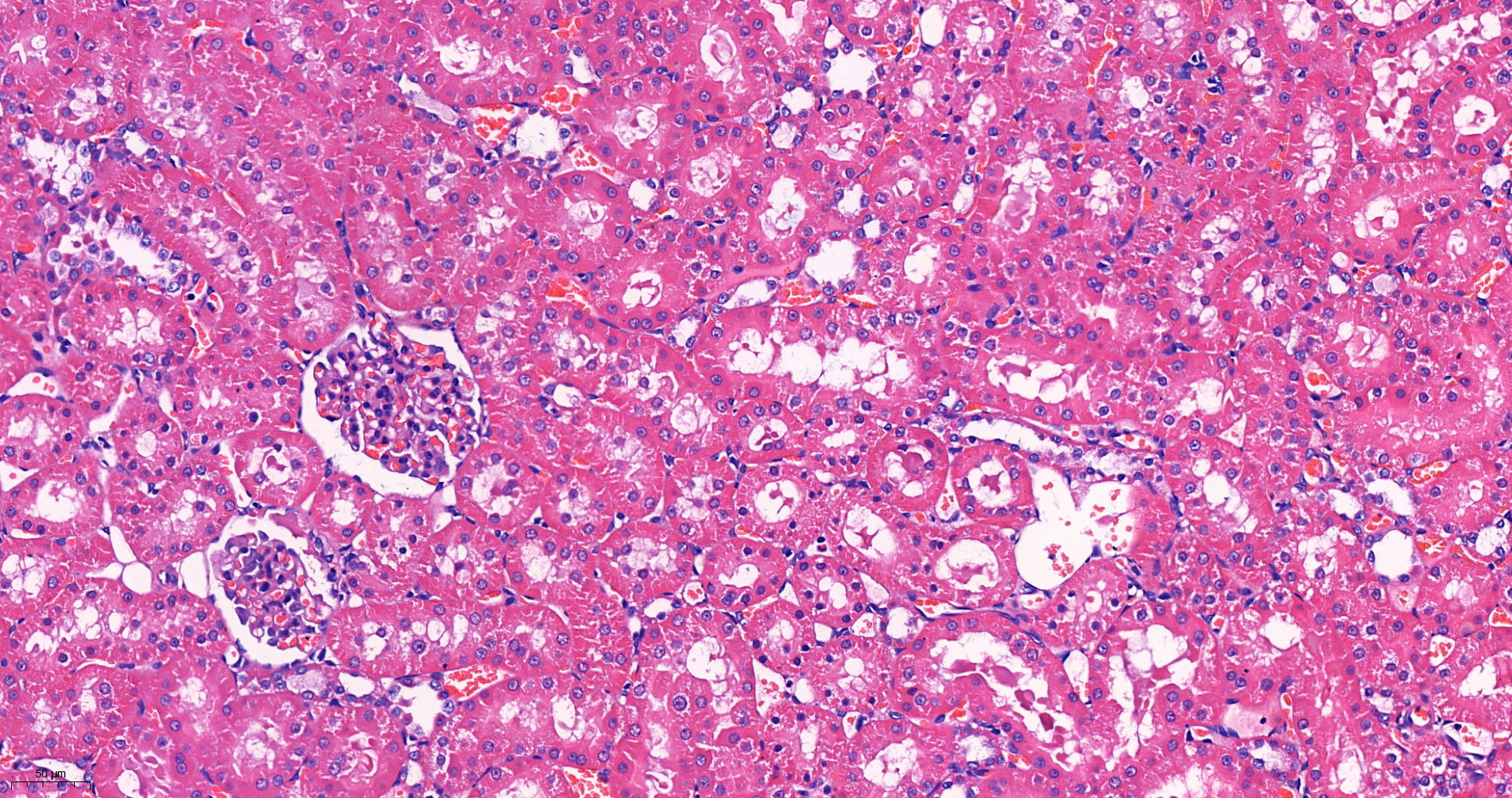

Supplement: Data S1 [file peerj-12-16709-s001.zip › Raw data/Figure 5/Figure 5A/CLP+postARI400X.jpg]

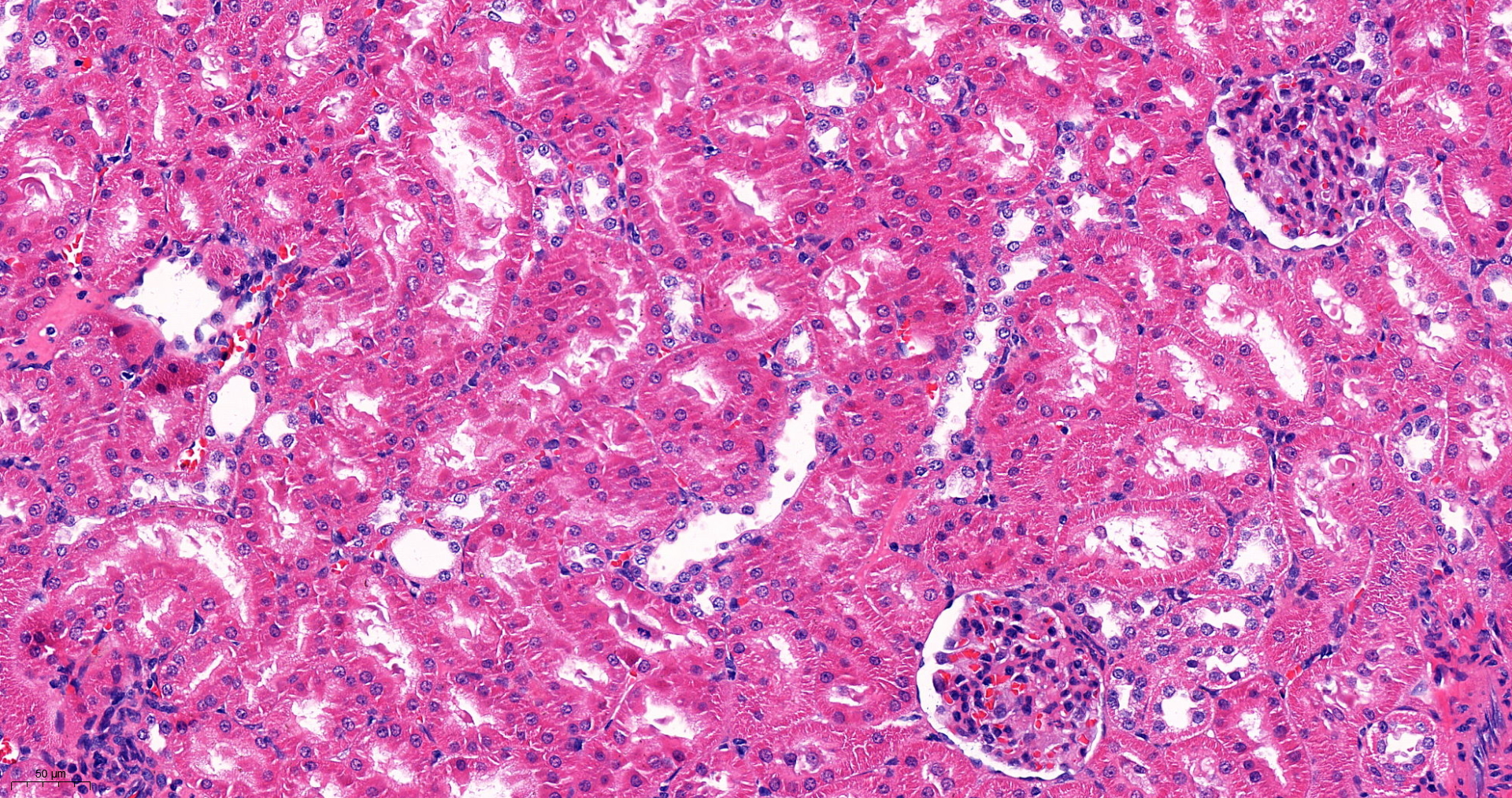

Supplement: Data S1 [file peerj-12-16709-s001.zip › Raw data/Figure 5/Figure 5A/CLP+pre-ARI400X.jpg]

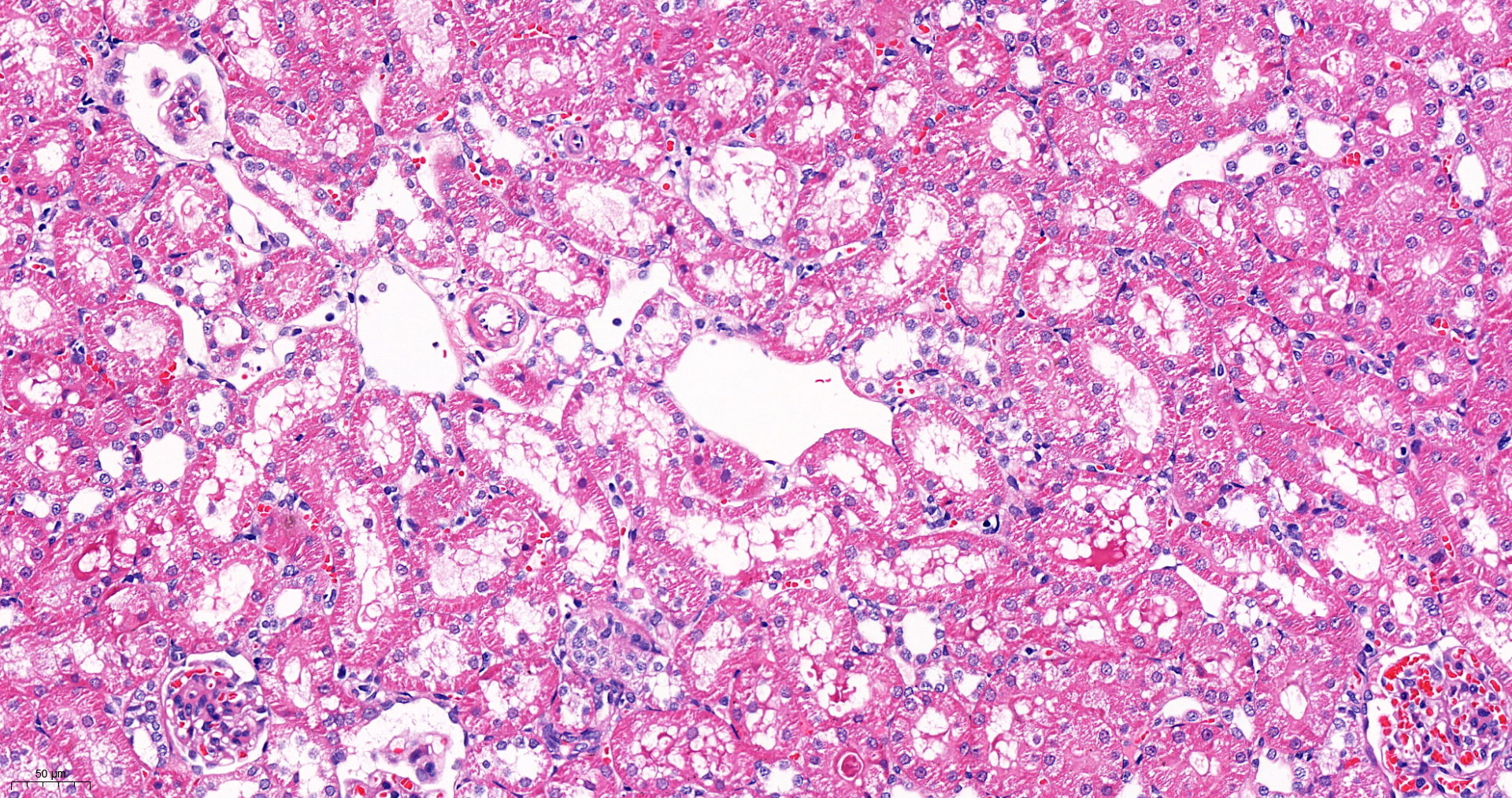

Supplement: Data S1 [file peerj-12-16709-s001.zip › Raw data/Figure 5/Figure 5A/CLP400X.jpg]

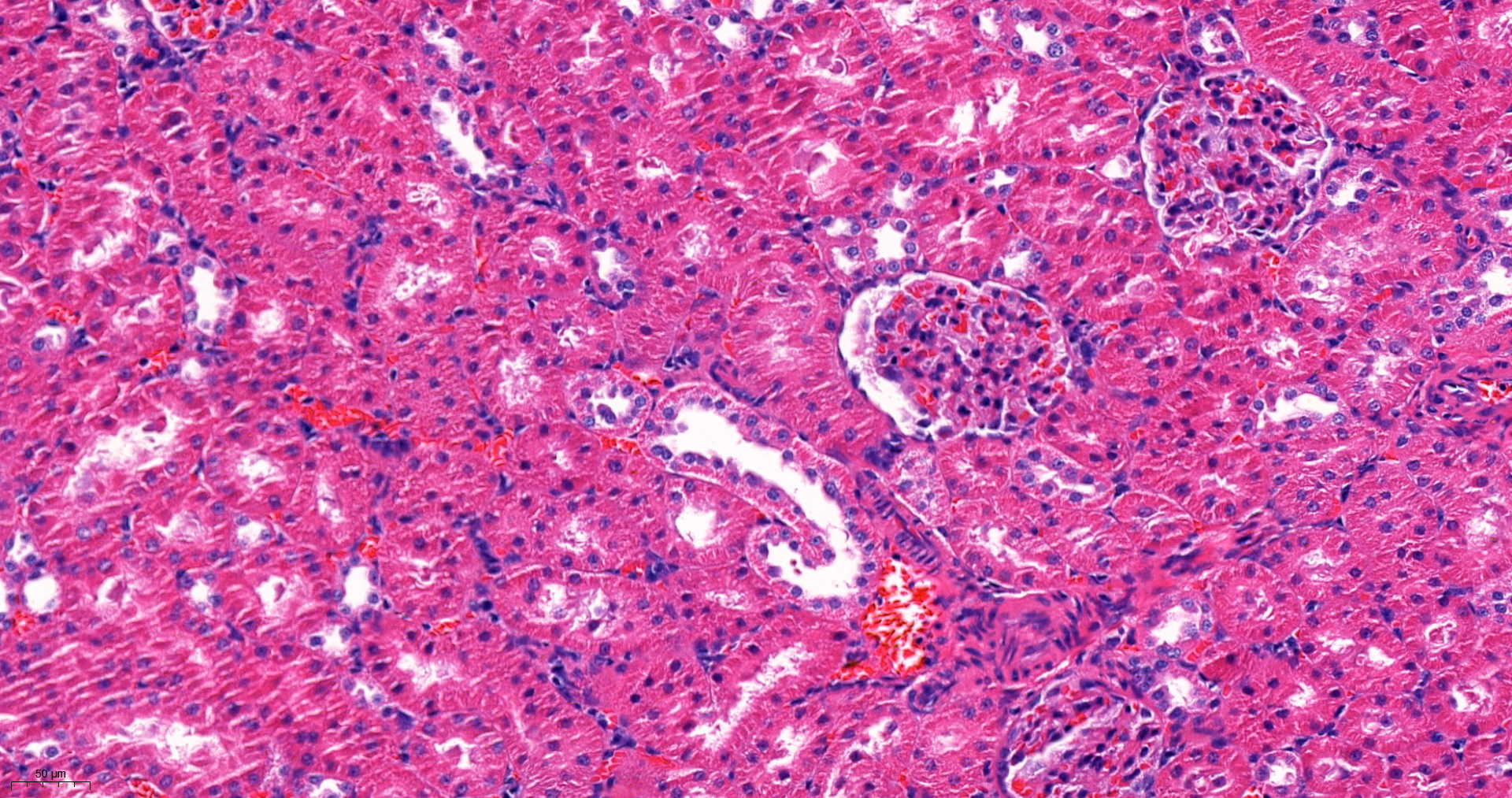

Supplement: Data S1 [file peerj-12-16709-s001.zip › Raw data/Figure 5/Figure 5A/sham400x.jpg]

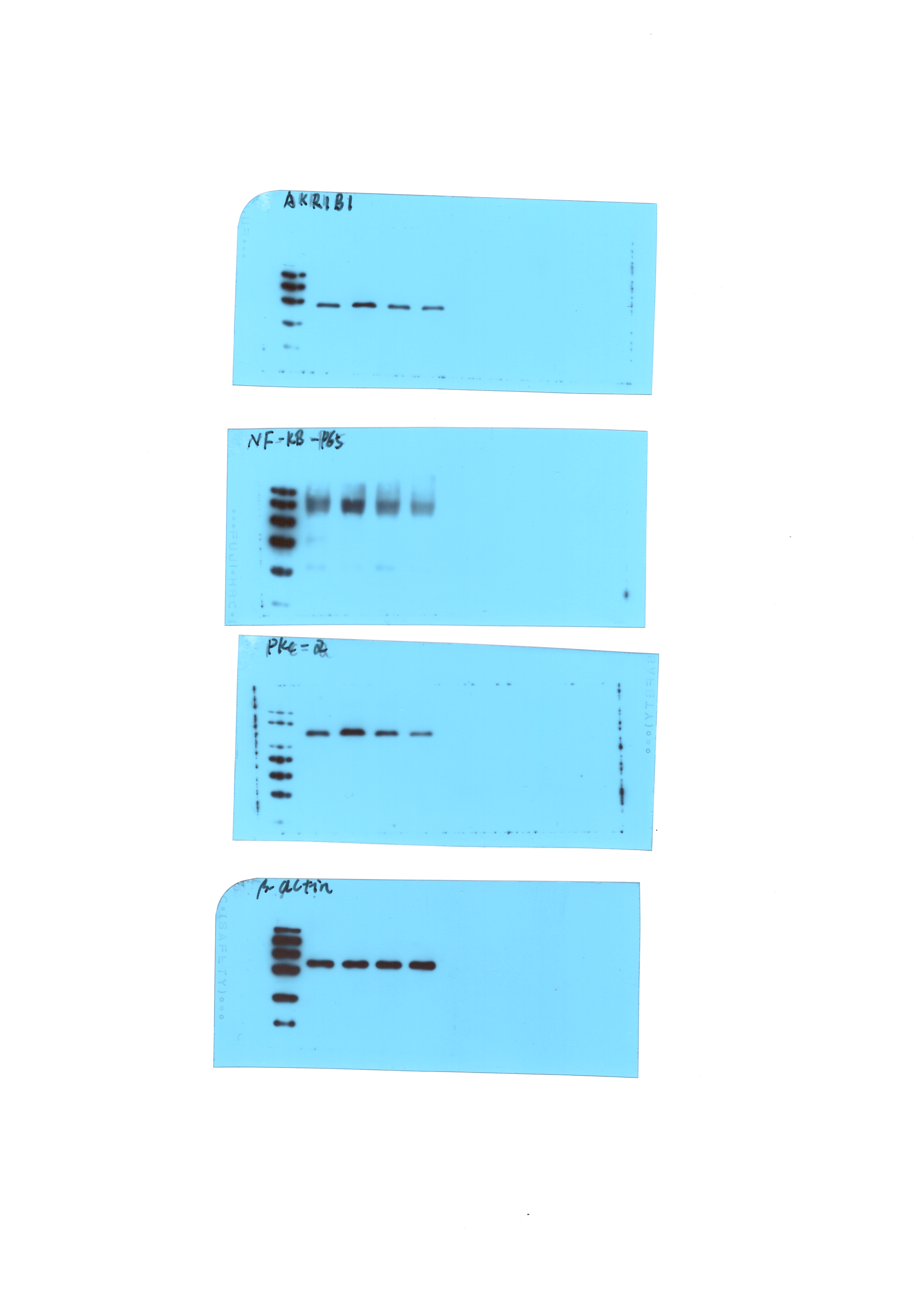

Supplement: Data S1 [file peerj-12-16709-s001.zip › Raw data/Figure 6/Figure 6B.tif]
