# Supplementary material for: Proteomics-based screening of AKR1B1 as a therapeutic target and validation study for sepsis-associated acute kidney injury
Source: PeerJ. 2024 Jan 2;12:e16709. doi: 10.7717/peerj.16709 (PMC10768659; doi:10.7717/peerj.16709)
Supplement: Supplemental Information 3 [file peerj-12-16709-s003.pdf]

ITEM TO CHECK	IMPORTANCE	CHECKLIST
**EXPERIMENTAL DESIGN**		
Definition of experimental and control groups	**E**	Line 260-261
Number within each group	**E**	Line 281-282
Assay carried out by core lab or investigator's lab?	D	Line 261-262
Acknowledgement of authors'contributions	D	N/A
**SAMPLE**		
Description	**E**	Line 260-261
Volume/mass of sample processed	D	Line 263
Microdissection or macrodissection	**E**	Line 263
Processing procedure	**E**	Line 262-265
If frozen - how and how quickly?	**E**	Line 262-265
If fixed - with what, how quickly?	**E**	N/A
Sample storage conditions and duration (especially for FFPE	**E**	Line 263
**NUCLEIC ACID EXTRACTION**		
Procedure and/or instrumentation	**E**	Line 264
Name of kit and details of any modifications	**E**	Line 264
Source of additional reagents used	D	Line 264
Details of DNase or RNAse treatment	**E**	Line 265-267
Contamination assessment (DNA or RNA)	**E**	Line 265-266
Nucleic acid quantification	**E**	Line 265-266
Instrument and method	**E**	Line 265-266
Purity (A260/A280)	D	Line 265-266
Yield	D	Line 265-266
RNA integrity method/instrument	**E**	Line 265-266
RIN/RQI or Cq of 3' and 5' transcripts	**E**	Line 265-266
Electrophoresis traces	D	N/A
Inhibition testing (Cq dilutions, spike or other)	**E**	Line 267-269
**REVERSE TRANSCRIPTION**		
Complete reaction conditions	**E**	Line 269-270
Amount of RNA and reaction volume	**E**	Line 269-270
Priming oligonucleotide (if using GSP) and concentration	**E**	Line 270
Reverse transcriptase and concentration	**E**	Line 268
Temperature and time	**E**	Line 270-271
Manufacturer of reagents and catalogue numbers	D	Line 267-269
Cqs with and without RT	D*	N/A
Storage conditions of cDNA	D	Line 271
**qPCR TARGET INFORMATION**		
If multiplex, efficiency and LOD of each assay.	**E**	Line 271
Sequence accession number	**E**	Line 283-284
Location of amplicon	D	Line 278-279
Amplicon length	**E**	Line 279
In silico specificity screen (BLAST, etc)	**E**	Line 279
Pseudogenes, retropseudogenes or other homologs?	D	N/A
Sequence alignment	D	Line 280
Secondary structure analysis of amplicon	D	N/A
Location of each primer by exon or intron (if applicable)	**E**	Line 279
What splice variants are targeted?	**E**	Line 279
**qPCR OLIGONUCLEOTIDES**		
Primer sequences	**E**	Line 279
RTPrimerDB Identification Number	D	N/A
Probe sequences	D**	N/A
Location and identity of any modifications	**E**	Line 279
Manufacturer of oligonucleotides	D	N/A
Purification method	D	N/A
**qPCR PROTOCOL**		
Complete reaction conditions	**E**	Line 275-278
Reaction volume and amount of cDNA/DNA	**E**	Line 272-273
Primer, (probe), Mg++ and dNTP concentrations	**E**	Line 275
Polymerase identity and concentration	**E**	Line 274
Buffer/kit identity and manufacturer	**E**	Line 274
Exact chemical constitution of the buffer	D	Line 274
Additives (SYBR Green I, DMSO, etc.)	**E**	Line 274
Manufacturer of plates/tubes and catalog number	D	Line 275
Complete thermocycling parameters	**E**	Line 275-277
Reaction setup (manual/robotic)	D	Line 275-277
Manufacturer of qPCR instrument	**E**	Line 272
**qPCR VALIDATION**		
Evidence of optimisation (from gradients)	D	N/A

Specificity (gel, sequence, melt, or digest)	**E**	Line 277-278
For SYBR Green I, Cq of the NTC	**E**	Line 279
Standard curves with slope and y-intercept	**E**	Line 281-283
PCR efficiency calculated from slope	**E**	Line 281-283
Confidence interval for PCR efficiency or standard error	D	Line 281-283
r2 of standard curve	**E**	Line 281-283
Linear dynamic range	**E**	Line 283
Cq variation at lower limit	**E**	Line 283-285
Confidence intervals throughout range	D	Line 283-285
Evidence for limit of detection	**E**	Line 283-285
If multiplex, efficiency and LOD of each assay.	**E**	Line 283-285
**DATA ANALYSIS**		
qPCR analysis program (source, version)	**E**	Line 281-283
Cq method determination	**E**	Line 281-283
Outlier identification and disposition	**E**	Line 281-283
Results of NTCs	**E**	Line 281-283
Justification of number and choice of reference genes	**E**	Line 281-283
Description of normalisation method	**E**	Line 281-283
Number and concordance of biological replicates	D	Line 287-288
Number and stage (RT or qPCR) of technical replicates	**E**	Line 287-288
Repeatability (intra-assay variation)	E	Line 285-286
Reproducibility (inter-assay variation, %CV)	D	Line 285-286
Power analysis	D	N/A
Statistical methods for result significance	**E**	Line 291-297
Software (source, version)	E	Line 291-297
Cq or raw data submission using RDML	**D**	N/A

Table 1. MIQE checklist for authors, reviewers and editors. All essential information (E) must be submitted with the manuscript. Desirable information (D) should be submitted if available. If using primers obtained from RTPrimerDB, information on qPCR target, oligonucleotides, protocols and validation is available from that source.

*: Assessing the absence of DNA using a no RT assay is essential when first extracting RNA. Once the sample has been validated as RDNA-free, inclusion of a no-RT control is desirable, but no longer essential.

**: Disclosure of the probe sequence is highly desirable and strongly encouraged. However, since not all commercial pre-designed assay vendors provide this information, it cannot be an essential requirement. Use of such assays is advised against.
